# Dressed *j*_eff_-1/2 objects in mixed-valence lacunar spinel molybdates

**DOI:** 10.1038/s41598-023-28656-3

**Published:** 2023-02-10

**Authors:** Thorben Petersen, Lilian Prodan, Korbinian Geirhos, Hiroyuki Nakamura, István Kézsmárki, Liviu Hozoi

**Affiliations:** 1 Institute for Theoretical Solid State Physics, Leibniz IFW Dresden, Helmholtzstr. 20, 01069 Dresden, Germany; 2 Experimental Physics V, Center for Electronic Correlations and Magnetism, University of Augsburg, 86135 Augsburg, Germany; 3 Present Address: Physik-Department, Technische Universität München, 85748 Garching, Germany; 4 Department of Materials Science and Engineering, Kyoto University, Kyoto, 606-8501 Japan

**Keywords:** Magnetic materials, Electronic properties and materials, Magnetic properties and materials, Electronic structure, Quantum chemistry

## Abstract

The lacunar-spinel chalcogenides exhibit magnetic centers in the form of transition-metal tetrahedra. On the basis of density-functional computations, the electronic ground state of an Mo_4_^13+^ tetrahedron has been postulated as single-configuration *a*_1_^2^ *e*^4^ *t*_2_^5^, where *a*_1_, *e*, and *t*_2_ are symmetry-adapted linear combinations of single-site Mo *t*_2g_ atomic orbitals. Here we unveil the many-body tetramer wave-function: we show that sizable correlations yield a weight of only 62% for the *a*_1_^2^ *e*^4^ *t*_2_^5^ configuration. While spin–orbit coupling within the peculiar valence orbital manifold is still effective, the expectation value of the spin–orbit operator and the *g* factors deviate from figures describing nominal *t*^5^ *j*_eff_ = 1/2 moments. As such, our data documents the dressing of a spin–orbit *j*_eff_ = 1/2 object with intra-tetramer excitations. Our results on the internal degrees of freedom of these magnetic moments provide a solid theoretical starting point in addressing the intriguing phase transitions observed at low temperatures in these materials.

## Introduction

Mixed valency, the presence of more than one oxidation state for a particular chemical element, is associated with very rich physics, in both molecular and solid-state systems. It can be intrinsic, as for example in stoichiometric magnetite Fe_3_O_4_^1^, or obtained through doping as in the colossal magneto-resistance manganese oxides (*R*_1-*x*_*A*_x_)MnO_3_ (*R* = rare-earth cation, *A* = alkali or alkaline-earth cation)^2^. It can also imply quasi-localized ligand-hole states, i. e., ligand mixed valency, as shown for underdoped cuprates^3,4^ or for the spin-Peierls compound NaV_2_O_5_^5,6^. Here we explore the mixed valence landscape in the molybdates GaMo_4_S_8_ and GaMo_4_Se_8_, lacunar-spinel quantum magnets displaying remarkable properties, e. g., modulated cycloidal and Néel-type skyrmion-lattice states with sub-10 nm periodicity that are coupled to ferroelectric polarization^7^, a plethora of ferroelectric domain boundaries and junctions^8^, and a cluster spin-glass phase^9^. Using advanced wave-function-based quantum chemical computational schemes, we show that the peculiar interplay of electronic correlations and spin–orbit interactions in these materials sets the stage for interesting physics and new insights: the dressing of a *j*_eff_ = 1/2 spin–orbit-coupled state^10^ with virtual valence space excitations. This dressing, or correlation cloud, is evidenced with the help of wave-function analysis and of observables such as the ground-state *g* factors and branching ratios. The use of the latter as indicators for ground-state correlations is possible due to the presence of sizable spin–orbit interactions: in their absence, information can be obtained by comparing ab initio and experimental data for e. g. the cohesive energy^11,12^. The outcome of magnetization measurements performed on GaMo_4_S_8_ supports the computational findings and calls for spectroscopic verification of other specific predictions we make, e. g., of *N*-particle excitation energies as probed by resonant inelastic x-ray scattering experiments.

## Results

### High-temperature electronic structure, dressed *j*_eff_ = 1/2 states

**Figure 1:**
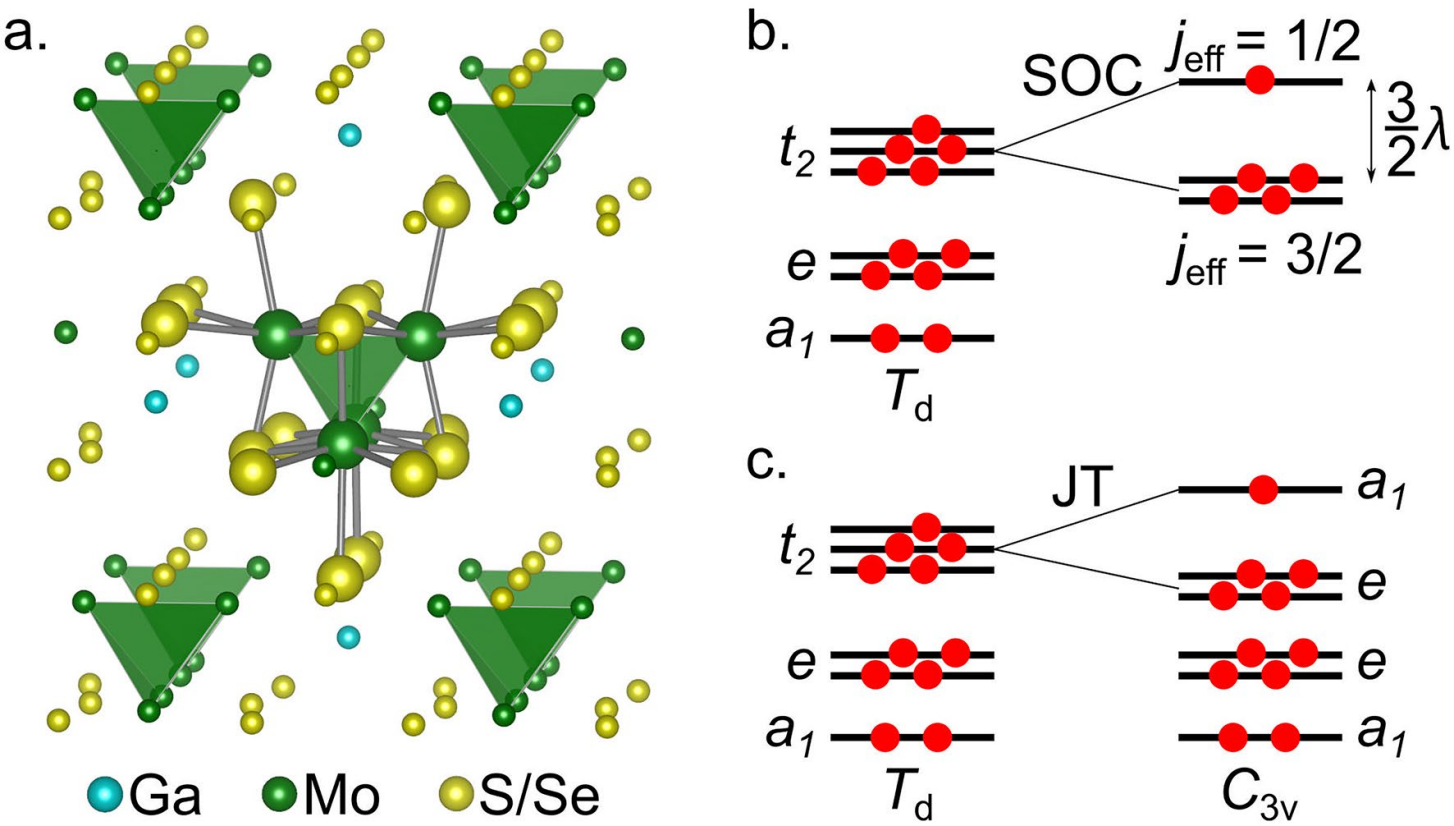
GaMo_4_*X*_8_ cluster model and molecular orbital schemes. (**a**) GaMo_4_S_8_/GaMo_4_Se_8_ cluster model. Mo_4_ tetrahedra are shown in green. Small atomic spheres indicate capped effective core potentials (cECPs). (Ga: blue, Mo: green, S/Se: yellow). (**b**) Molecular-orbital diagram for the Mo_4_ cluster in high-temperature, cubic *T*_d_ point group with spin–orbit coupling (SOC) and low-temperature, rhombohedral *C*_3v_ symmetry as a result of Jahn–Teller (JT) distortion.

Spinels are generically described by the chemical formula *AM*_2_*X*_4_. The *M* sites define a pyrochlore lattice, each of those ions being encapsulated within an *X*_6_ octahedral cage of nearest-neighbor chalcogenide ligands. Magnetite, for example, belongs to this class of systems, with Fe ions at both *A* and *M* sites^1^. In *lacunar* spinels, the *A* sites have half-integer occupation. With every second *A* cation removed, nearest-neighbor *M*_4_ tetrahedra of the initial pyrochlore structure experience different surroundings; structural relaxation in the new setting yields a so called “breathing” pyrochlore lattice. The simplest way of representing that is as a *fcc* network of *M*_4_ clusters (or of *M*_4_*X*_16_ units), see Fig. 1a; adjacent *M*_4_ entities share no *M* site.

Prior electronic-structure calculations based on density functional theory^13,14^ point to a single-tetramer molecular-orbital-like diagram with a set of *a*_1_, *e*, and *t*_2_ levels at the top of the occupied orbital space, where the notations correspond to *T*_d_ point-group symmetry (see Fig. 1b). In GaMo_4_S_8_ and GaMo_4_Se_8_ there are formally 2.75 electrons per transition-metal (TM) site (i. e., 4 × 2.75 = 11 *d* electrons per Mo_4_ cluster). Since the *t*_2_ levels come at higher energy as compared to the *a*_1_ and *e* components, the basic single-tetrahedron electronic structure in GaMo_4_*X*_8_ would then imply filled *a*_1_ and *e* states and one hole within the *t*_2_ sector. However, given the results of state-of-the-art quantum chemical calculations on related “clustered” compounds, both d-^15–18^ and p-electron^19^ based, there are reasons to believe that the picture presently put forward for the single-tetramer electronic structure in GaMo_4_*X*_8_ is somewhat too sketchy.

For insights into the *correlated* electronic structure of Mo_4_^13+^ units in GaMo_4_S_8_ and GaMo_4_Se_8_, we adopt the embedded-cluster quantum chemical approach described in Ref.^18^ and the lattice parameters and atomic positions determined by Powell et al*.* for GaMo_4_S_8_^20^ and Routledge et al*.* for GaMo_4_Se_8_^21^. An [Mo_4_*X*_16_]^19-^ cluster was in each case embedded within a large array of point charges created with the Ewald program^22,23^; capped effective core potentials (cECPs) were assigned to the 98 closest atoms. Further details on the embedding procedure are provided in the Supplemental Material (SM-1). The more general theoretical frame is mapped in Refs.^11,24^.

The nature of the ground state and the low-energy excitation spectrum of a Mo_4_ tetrahedron were first analyzed for the high-temperature (HT) structure, in both GaMo_4_S_8_ and GaMo_4_Se_8_. The leading ground-state configuration is *a*_1_^2^ *e*^4^ *t*_2_^5^ *t*_1_^0^ *t*_2_^0^ (c.f. Figure 1b), but for a “converged” CAS(11e,12o) orbital space it comes with a weight of only 62% in the CASSCF ground-state wave-function (see Supplemental Material SM-3 for the active space orbitals and Supplementary Tables S1 and S2 in Supplemental Material SM-4 for the excited state configurations). The remaining configurations are mostly double excitations, e. g., *a*_1_^2^ *e*^3^ *t*_2_^4^ *t*_1_^1^ *t*_2_^1^; there is a multitude of those, each contributing to the overall wave-function with a weight of less than 5%.

These findings do not seem unusual for this class of materials: important multiconfigurational effects, i. e., a weight of the *a*_1_^2^ *e*^4^ *t*_2_^1^ leading electron configuration of less than 70%, were also found in the related 4*d* lacunar spinel GaNb_4_Se_8_^18^. The competition between different electronic configurations seems to be even sharper in the vanadium analogue^17,25^.

**Figure 2:**
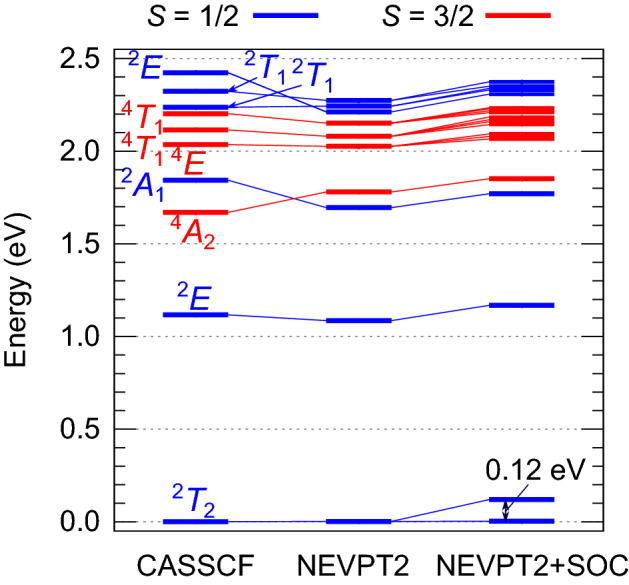
Low-energy excitation energies for high-temperature GaMo_4_S_8_. The spectrum was calculated using an [Mo_4_S_16_]^19-^ embedded cluster model (CAS(11e,12o)). Both CASSCF and NEVPT2 values (also with SOC for the latter) are given in eV. Four quartets and six doublets were included in the state-averaging procedure. Notations according to *T*_d_ point group symmetry are used.

Spin–orbit coupling (SOC) is effective for the multiconfigurational ^2^*T*_2_ ground-state, giving rise to a low-lying spin–orbit doublet and a spin–orbit quartet with an excitation energy of about 0.12 eV (see Figs. 1b, 2). Yet, different from the (…*a*_1_^2^ *e*^4^) *t*_2_^5^ leading ground state configuration, unambiguously described in terms of *S* = 1, *L*_eff_ = 1/2, and *j*_eff_ = 1/2 quantum numbers^10^, the multiconfigurational case entails more subtle nuances. This is discussed in the following paragraphs.

As depicted in Fig. 2, further excited states follow at energies exceeding 1 eV. Different from the ground ^2^*T*_2_ term, spin–orbit interactions play a less important role for the higher-lying multiplets. But noticeable modifications occur post-CASSCF for the latter, at the NEVPT2 level; the sequence of the ^4^*A*_2_ and ^2^*A*_1_ terms, for instance, is different in NEVPT2 as compared to CASSCF. Comparing the NEVPT2 energies in GaMo_4_S_8_ and GaMo_4_Se_8_ (see Supplementary Material SM-4), it is seen that the excitation spectra are rather similar, somewhat scaled down in the latter compound. This can be attributed to having longer Mo-Se bonds in GaMo_4_Se_8_, i. e., weaker ligand fields.

To quantify the strength of spin–orbit interactions, the SOC constant *λ* was determined from minimal active space calculations. For such a CAS(5e,3o), which only incorporates the (…*a*_1_^2^ *e*^4^) *t*_2_^5^ configuration, the *j*_eff_ = 3/2 quartet is split by 100 meV from the *j*_eff_ = 1/2 ground-state term, yielding a SOC constant $\lambda_{{{\text{SOC}}}}^{{{\text{Mo}}_{4} }} = 67\; {\text{meV}} = 538\;{\text{ cm}}^{ - 1}$. This value lies close to the SOC constant estimated for the (…*a*_1_^2^ *e*^4^) *t*_2_^1^ compound GaNb_4_Se_8_, $\lambda_{{{\text{SOC}}}}^{{{\text{Nb}}_{4} }} = 516 \;{\text{cm}}^{ - 1}$^18^. Compared to a Mo^+^ single-ion value $\lambda_{{{\text{SOC}}}}^{{{\text{Mo}}^{ + } }} = 630 \;{\text{cm}}^{ - 1}$^10^, the SOC parameter is lower in GaMo_4_Se_8_, due to the more delocalized character of lacunar-spinel *t*_2_ electrons – those orbit in lacunar spinels a four-site unit, not a single TM ion. The fact that the splitting between the lowest spin–orbit states increases when using larger active spaces (from 0.1 to 0.12 eV, see Fig. 2) has to do with electron correlations that bring in multiconfigurational physics; such effects were not addressed in earlier computations based on density functional theory^14,26^.

A peculiar feature of nominal *j*_eff_ = 1/2 ground states is free-electron-like *g* factors^10^, even if *L*_eff_ = 1. On the basis of the active space employed for the calculations [i. e., CAS(11e,12o)], ground-state *g* factors of 2.18 are obtained. The sizable deviation from 2 has to do with contributions of other electron configurations to the multiconfigurational ground-state wave-function, i. e., dressing with intra-tetramer excitations. For comparison, magnetic susceptibility measurements indicate a *g* value of 2.37 (see Supplementary Material SM-5). The somewhat larger experimental estimations hint towards additional vibronic effects^27,28^ not included in the rigid cluster model.

Another quantity that can provide evidence for such dressing is the branching ratio (BR) in *L*-edge x-ray absorption spectroscopy (XAS), i. e., the ratio of the integrated intensities of the *L*_3_ and *L*_2_ absorption edges. It is directly related to the ground-state expectation value ${\mathcal{Z}}$ of the spin–orbit Hamiltonian and can be compactly expressed as ${\text{BR}} = \left( {2 + r} \right)/\left( {1 - r} \right)$^29–31^, where $r = {\mathcal{Z}}/n_{{\text{h}}}$, $n_{{\text{h}}}$ is the number of holes in the valence shell, and for a given *LS* term ${\mathcal{Z}} = \lambda^{ - 1} {\mathcal{H}}_{{{\text{SO}}}} = {\mathbf{L}} \cdot {\mathbf{S}}$. ${\mathcal{Z}}$ can be alternatively written as^30–32^

1 $${\mathcal{Z}} = \lambda^{ - 1} \left( {E_{{{\text{LS}}}}^{0} - E_{{{\text{LSJ}}}}^{0} } \right),$$

where $E_{{{\text{LS}}}}^{0}$ and $E_{{{\text{LSJ}}}}^{0}$ are ground-state eigenvalues without and with SOC, respectively. $E_{{{\text{LSJ}}}}^{0}$ may depend on both first- and second-order SOCs.

As an example, for a nominal *j*_eff_ = 1/2 spin–orbit ground state in *t*_2g_^5^ iridates^33,34^ ($n_{{\text{h}}} = 5$), values ${\mathcal{Z}}$ = 1 and BR = 2.75 are expected^35,36^; in contrast, for vanishing valence-shell spin–orbit interactions, ${\mathcal{Z}}$ → 0 and BR → 2 are obtained. For the lacunar-spinel molybdates, using the minimal active space in the CASSCF calculation (five electrons within the three *t*_2_ cluster orbitals), we find indeed ${\mathcal{Z}}$ = 1 (see Table 1, first line). Slight reduction of the NEVPT2 minimal-CAS ${\mathcal{Z}}$ to 0.99 indicates the admixture of other electron configurations in the NEVPT2 treatment. Proper description of multiconfigurational physics requires however larger active spaces: enlarging the active space to CAS(11e,12o), ${\mathcal{Z}}$ increases to 1.2–1.3, even when only the lowest-lying ^2^*T*_2_ term is included in the spin–orbit part of the computation. The difference between the large-CAS ${\mathcal{Z}}$, $\approx$ 1.2, and the minimal-CAS ${\mathcal{Z}}$ of 1 is an indicator for the amount of dressing of the *j*_eff_ = 1/2 object with intra-tetramer electronic excitations. Interestingly, while in e. g. *t*_2g_^5^ iridates or rhodates this difference mainly arises from strong second-order SOCs^32,35,36^, here those are inactive at HTs. ${\mathcal{Z}}$ being smaller and $n_{{\text{h}}}$ larger than in *t*_2g_^5^ iridates, the BR is expected to be less than $\approx$ 2.5 in the molybdate lacunar spinels, which can be verified by XAS measurements.

**Table 1:** ${\mathbf{\mathcal{Z}}}$ values in GaMo_4_S_8_ and GaMo_4_Se_8_.

Number of states	Active space	[Mo_4_S_16_]^19-^		[Mo_4_Se_16_]^19-^	
		CASSCF	PT2	CASSCF	PT2
1D (^2^*T*_2_)	CAS(5e,3o)	1.00	0.99	1.00	0.99
1D (^2^*T*_2_)	CAS(11e,12o)	1.28	1.27	1.26	1.22
6D, 4Q	CAS(11e,12o)	1.26	1.23	1.25	1.22

The ${\mathcal{Z}}$ values are given for different types of reference active spaces and different number of states in the spin-orbit treatment. The abbreviations refer to doublet (D) and quartet (Q) states. PT2 stands for NEVPT2.

### Low-temperature electronic structure, $\tilde{S}$ = 1/2 pseudo-spins

**Figure 3:**
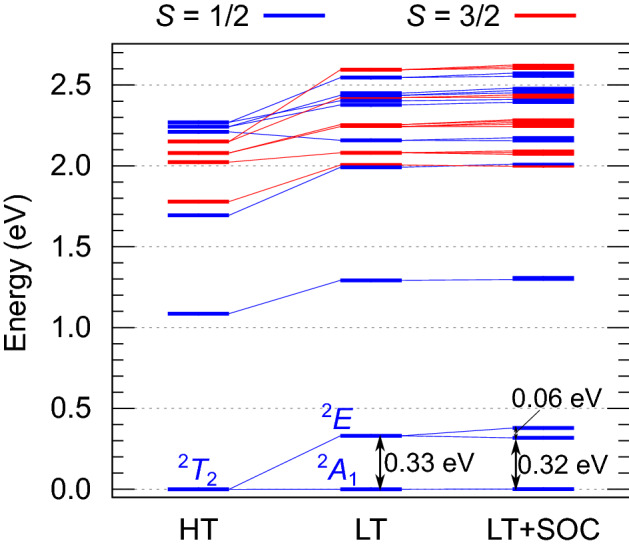
Influence of Jahn–Teller distortion on the low-energy excitation spectrum of GaMo_4_S_8_. Both high-temperature (HT) and low-temperature (LT) energies are given for the [Mo_4_S_16_]^19-^ cluster model (NEVPT2(11e,12o)), including SOC for the latter. Six quartets and ten doublets were included in the state-averaging procedure.

At temperatures of about 50 K, the GaMo_4_*X*_8_ lacunar spinels undergo a phase transition from cubic (*F*$\overline{4}3$m) to rhombohedral (*R*3*m*) space group^13^, with compression of the [Mo_4_*X*_4_]^5+^ tetrahedra along the cubic [111] direction, i. e., along a line connecting opposite Mo and *X* vertices^13,37,38^. This polar distortion gives rise to axial magnetic anisotropy along [111]. The effects of structural distortions as reported by Powell et al*.* for GaMo_4_S_8_^20^ and by Routledge et al*.* for GaMo_4_Se_8_^21^ were analyzed on the basis of CASSCF + NEVPT2 computations and orbital active spaces as employed for the HT phase (see Fig. 3 for GaMo_4_S_8_ and Supplementary Material SM-4 for GaMo_4_Se_8_).

Trigonal crystal-field splittings as large as 0.33 eV and 0.24 eV for GaMo_4_S_8_ and GaMo_4_Se_8_, respectively, are found to occur within the *t*_2_ manifold, as illustrated by the splitting of the HT ground ^2^*T*_2_ state into ^2^*A*_1_ and ^2^*E* terms. Obviously, this renders the *j*_eff_ = 1/2 picture inadequate. Yet, the effect of SOC is not negligible—three distinct Kramers doublets are found in the spin–orbit calculations, with relative NEVPT2 energies of 0, 0.32/0.23, and 0.38/0.29 eV. From a magnetic point of view, the pertinent effective picture for the Mo_4_ tetrahedra is therefore that of $\tilde{S}$ = 1/2 moments with significant angular-momentum contribution, in accordance with susceptibility measurements^39^. This is further seen in the structure of the ab initio low-*T* (LT) *g* factors (Table 2), where $g_{||}$ and $g_{ \bot }$ refer to directions parallel and perpendicular to the rhombohedral axis (the [111] axis in cubic setting), respectively. A similar relation, $g_{||} < g_{ \bot }$, was found for positive trigonal/tetragonal splittings in RuCl_3_^40^ and Ba_2_IrO_4_^32,41^.

**Table 2:** Calculated *g* factors for LT [Mo_4_*X*_16_]^19-^ (*X* = S, Se) cluster models (CAS(11e,12o)).

Method	LT [Mo_4_S_16_]^19-^		LT [Mo_4_Se_16_]^19-^	
	$g_{||}$ ^*a*^	$g_{ \bot }$	$g_{||}$ ^*a*^	$g_{ \bot }$
CASSCF	1.87	2.43	1.76	2.56
NEVPT2	1.80	2.53	1.56	2.69
Exp.		2.54		

^a^$g_{||}$ refers to the cubic [111] axis (see also Fig. 4).

**Figure 4:**
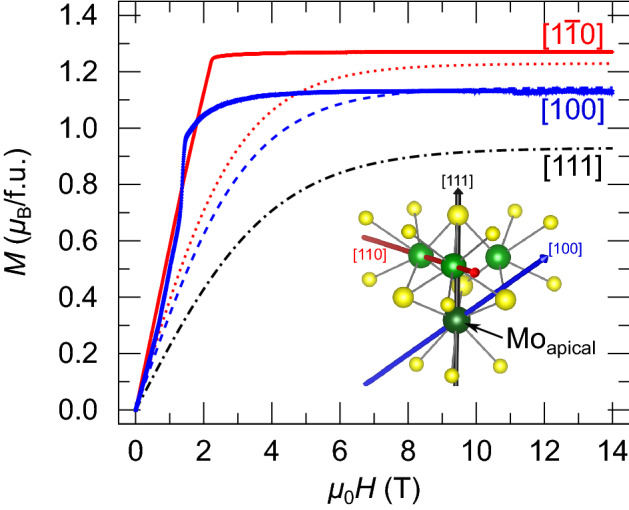
Magnetization curves for LT GaMo_4_S_8_. Measured (thick full lines) and simulated (dashed lines) curves were obtained at a temperature of 2.5 K. [110] (red), [100] (blue), and [111] (black) directions are given in the cubic setting. The simulated curve is obtained for a [Mo_4_S_16_]^19-^ embedded cluster (CASSCF(11e,12o)).

In order to experimentally verify the predicted type of axial anisotropy, characterized by $g_{||} /{\text{g}}_{ \bot } < 1$, we measured the magnetization of LT GaMo_4_S_8_ along two non-equivalent directions, the [110] and [100] axes shown in Fig. 4. The [110] direction is perpendicular to the [111] rhombohedral axis, hence the corresponding saturation magnetization directly yields ${g}_{ \bot }^{{{\text{exp}}}}$. Measurements with field applied along the [100] axis yield a combination of *g* factors, namely $\sqrt {g_{||}^{2} \cos^{2} \left( {54.7^\circ } \right) + g_{ \bot }^{2} \sin^{2} \left( {54.7^\circ } \right)}$. Below 2 T, the low-temperature phase of GaMo_4_S_8_ shows a sequence of meta-magnetic transitions between different modulated magnetic states, including the skyrmionic lattice state^7^, which is evidenced by anomalies in this region. These magnetic states are not the focus of the present work; instead, we study the high-field magnetization, with special emphasis on the anisotropy of the saturation magnetization *μ*_sat_. That allows us to determine the anisotropy of the *g* tensor. For both field directions, the measured magnetization is clearly saturated above ≈6 T. We find a tiny (≈1%) decrease of the magnetization at higher fields, originating from a small diamagnetic contribution of the sample holder. This is visible only when the magnetization of the crystal is fully saturated.

For comparison, magnetization curves computed on the basis of the CASSCF(11e,12o) *g* factors^42^ are also shown. At low fields between 1 and 4 T the simulated curves underestimate the measured magnetization, likely because of the single-tetrahedron model for which inter-tetrahedral magnetic interactions are absent. However, the experimental and simulated saturation magnetization values are in good agreement above 6 T, with $\mu_{{{\text{sat}}}}^{{\left[ {110} \right]}} = 1.27 \mu_{{\text{B}}}$ and $\mu_{{{\text{sat}}}}^{{\left[ {100} \right]}} = 1.12 \mu_{{\text{B}}}$. Since our calculated values are fully ab initio (with no ad hoc parameters in the underlying theory), small deviations can be assigned to structural differences present in experiment and embedded-cluster computations (see Supplemental Material SM-6).

## Conclusions

With the help of ab initio quantum chemical calculations for both high- and low-temperature structural configurations, we shed light onto the nature of magnetic moments in mixed valence GaMo_4_S_8_ and GaMo_4_Se_8_. The quantum chemical computational data is referenced against low-temperature magnetization measurements. Our study documents the dressing of a bare *j*_eff_ = 1/2 object^10^ with valence space excitations. We analyzed in this regard the wave-function itself and observables such as the ground-state *g* factors and the so-called branching ratios. The essential physics refers to genuine electron correlation effects, different from the case of layered *t*_2g_^5^ iridium oxides^33,34^ where the branching ratios and ground-state *g* factors deviate from values corresponding to nominal *j*_eff_ = 1/2 moments due to *j*_eff_ 1/2–3/2 mixing and second-order spin–orbit couplings in the presence of lower-symmetry crystal/ligand fields^32,41^. The dressing, or correlation cloud, evidenced here has to do with the ground state of a Mo_4_ tetramer, different from the quasiparticle concept utilized for excitations in solids. Its internal degrees of freedom determine the polar properties of the material, although a comprehensive description requires additionally an adequate treatment of vibronic couplings and inter-tetramer interactions.

## Methods

All calculations were performed with the Orca program package, v5.0^43^. Near-degeneracy correlation effects were accounted for by complete active space self-consistent field (CASSCF) theory^44,45^ while additional dynamical correlations were described by *N*-electron valence second-order perturbation theory (NEVPT2)^46^. In the framework of the Douglas-Kroll-Hess (DKH) approximation^47^, the all-electron SARC-DKH-TZVPP^48^ basis set for Mo and the DKH-DEF2-TZVPP^49^ basis set for S/Se were used to treat SOC. To the cECPs, pseudopotentials of Andrae et al*.*^50^ for Mo, Bergner et al*.*^51^ and Leininger et al*.*^52^ for Ga and S/Se were assigned. Additional computational details are given in the Supplemental Material (SM-2). For direct connection with experimental results, the magnetization of a GaMo_4_S_8_ single crystal grown by the flux method^53^ was measured at 2.5 K in fields up to 14 T using a Quantum Design MPMS3.

## Supplementary Information

Supplementary Information 1.

Supplementary Information 2.

## Data Availability

The datasets generated during and/or analysed during the current study are available from the corresponding author on reasonable request.
