# Supplementary material for: Dressed jeff-1/2 objects in mixed-valence lacunar spinel molybdates
Source: Sci Rep. 2023 Feb 10;13:2411. doi: 10.1038/s41598-023-28656-3 (PMC9918447; doi:10.1038/s41598-023-28656-3)
Supplement: Supplementary file 2 — Supplementary Information 2. [file 41598_2023_28656_MOESM2_ESM.pdf]

Supplementary Material:

Dressed j_{eff} = 1/2 pseudospins in mixed valence lacunar spinel molybdates

Thorben Petersen,*[,a] Lilian Prodan,[b] Korbinian Geirhos,[b,c]
Hiroyuki Nakamura,[d] István Kézsmárki,*[,b] and Liviu Hozoi*[,a]

[a] Institute for Theoretical Solid State Physics, Leibniz IFW Dresden, Helmholtzstr. 20, D-01069 Dresden, Germany. Tel: +49 4659 1837; E-mail: t.petersen@ifw-dresden.de, l.hozoi@ifw-dresden.de

[b] Experimental Physics V, Center for Electronic Correlations and Magnetism, University of Augsburg, D-86135 Augsburg, Germany; E-mail: istvan.kezsmarki@physik.uni-augsburg.de

[c] Physik-Department, Technische Universität München, D-85748, Garching, Germany.

[d] Department of Materials Science and Engineering, Kyoto University, Kyoto 606-8501, Japan.

Table of Contents

SM-1	Embedded Cluster Protocol	1
SM-2	Computational Details	1
SM-3	Active Space Set-up	2
SM-4	Excited State Energies of $GaMo_4S_8$ and $GaMo_4Se_8$	3
	- Low-energy excitation energies of high-temperature (HT) phase	3
	- Low-energy excitation energies of low-temperature (LT) phase	4
SM-5	Susceptibility Measurements on $GaMo_4S_8$	6
SM-6	Different Low-temperature (LT) $GaMo_4S_8$ Structures	7
SM-7	Orca Input	8
	Supplementary References	10

SM-1. Embedded Cluster Approach

Both $GaMo_4S_8$ and $GaMo_4Se_8$ crystallize in a cubic *fcc* lattice with the space group $F\bar{4}3m$ under high-temperature.[1] The main building unit is represented by a breathing pyrochlore lattice of the Mo sites, with alternating Mo_4 tetrahedra connected via corners. The electronic structure of this particular Mo_4 unit was studied using an embedded cluster model in a similar manner as described previously for $GaNb_4Se_8$/$GaTa_4Se_8$.[2] Starting from the respective experimentally determined structures for $GaMo_4S_8$[3] and $GaMo_4Se_8$[4], a large array of more than 10 000 point charges was created using the EWALD program.[5,6] Here, initial charges of +3, +2.75, -1.5 and -2 for Ga, Mo, inner and outer *X* (with *X* = S, Se), respectively, were assumed. The term "inner" atoms refer to anions forming the main $[Mo_4S_4]$/$[Mo_4Se_4]$ magnetic unit, while "outer" atoms are the remaining anions bonding to Ga. Within a small sphere around the central cluster model, these initial charges are fixed, while those in the extended lattice are optimized to satisfy the Madelung convergence criterion for the whole lattice. Consequently, an $[Mo_4X_{16}]^{19-}$ cluster model was embedded in the center of this point charge lattice through assigning capped effective core potentials (cECPs) to the 98 closest atoms (see Fig. 1 in the manuscript). Additionally, it was ensured that the electrostatic potential fitted charges of the quantum cluster closely resemble the initial charges given above.[7,8] The coordinates of point charge field and quantum cluster are given in the separate spreadsheets (zip file).

SM-2. Computational Details

The strongly correlated electronic structure within the Mo_4 unit was investigated using the complete active space self-consistent field (CASSCF) method.[9] Missing dynamical correlation was accounted for by the *N*-electron valence 2nd order perturbation theory (NEVPT2) method[10] with all internal orbitals correlated. Besides, the relativistic Douglas-Kroll-Hess (DKH) approximation[11] was enabled and the all-electron SARC-DKH-TZVPP[12] basis set for Mo and DKH-DEF2-TZVPP[13] basis set for S/Se were used to properly treat SOC. For further efficiency, the RIJCOSX approximation with automatic generation of Coulomb and Exchange auxiliary basis sets[14] for Mo and the DEF2/J[15] and DEF2-TZVPP/C[16] auxiliary basis sets for S/Se was applied. Those auxiliary basis functions were decontracted to be used together with the DKH method. Finally, the cECPs were modeled through pseudopotentials of Andrae *et al.*[17] for Mo and Bergner *et al.*[18] and Leininger *et al.*[19] for Ga and S. Calculations were performed with the ORCA program package, v5.0.[20]

SM-3. Active Space Set-up

In accordance to our previous work,[2] quasi-restricted orbitals (QROs) from a preceding unrestricted Kohn-Sham (UKS) DFT calculation of $[Mo_4S_{16}]^{17-}$ and $[Mo_4Se_{16}]^{17-}$ embedded cluster models, respectively, in high-spin configuration $S = 1.5$ (with two electrons removed, i.e. $a_1^2\, e^4\, t_2^3$ configuration, using the B3LYP functional[21-24] were generated. Subsequently, these orbitals were used for the actual CASSCF calculation, now correctly accounting for the Mo_4^{13+} unit with $4 \times (6\text{-}3.25) = 11$ electrons. The final CAS(11e,12o) natural orbitals of the $[Mo_4X_{16}]^{19-}$ embedded cluster model are depicted in Figure S1. The natural orbitals of the active space can be assigned according to T_d point group symmetry to a leading configuration of $a_1^2\, e^4\, t_2^5\, t_1^0\, t_2^0$ (62%) in the ground state.

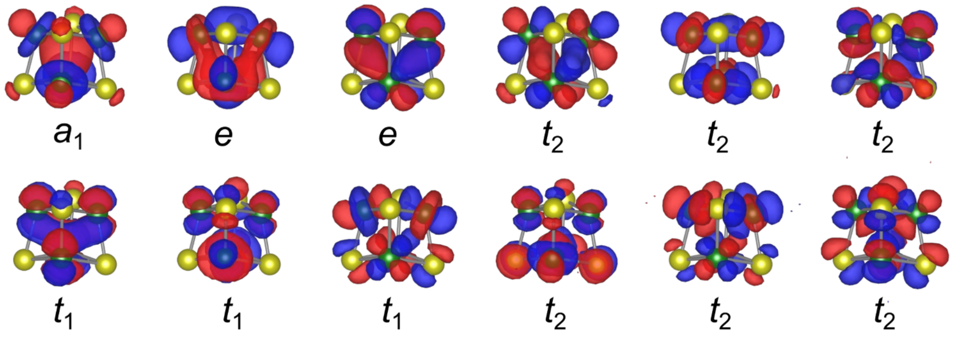

Figure S1. Twelve-orbital active space in molecular orbital representation. Notation according to T_d point group symmetry.

SM-4. Excited State Energies of $GaMo_4S_8$ and $GaMo_4Se_8$

Low-energy excitation energies of high-temperature (HT) phase

In the following Tables S1 and S2, the low-energy excitation energies of $GaMo_4S_8$ and $GaMo_4Se_8$ in the high-temperature (HT) cubic $F\bar{4}3m$ phase on CASSCF(11e,12o) and NEVPT2 level of theory are listed. For both, also the influence of spin-orbit coupling (SOC) on the excited state energies are given.
Focusing on $GaMo_4S_8$, the $^2T_2$ ground state is split by SOC into j = 1/2 and j = 3/2 effective states by 0.12 eV. The magnitude of this splitting is similar in CASSCF and NEVPT2, since the underlying wavefunction is not changed by NEVPT2. As a further effect of SOC, the following low-energy excitation energies are increased by about 0.08 eV through SOC.

Table S1. Low-energy excitation energies (eV) of high-temperature (HT) $[Mo_4S_{16}]^{19-}$ (CAS(11e,12o)). Four quartets and six doublets were included in the state-averaging procedure. Notation according to T_d point group symmetry.

State	Leading config. (weight)	E_{CASSCF}	$E_{CASSCF+SOC}$	E_{NEVPT2}	$E_{NEVPT2+SOC}$
$^2T_2$	$a_1^2\, e^4\, t_2^5\, t_1^0\, t_2^0$ (62%)	0.00	0.00, 0.12 (j = 1/2, j = 3/2)	0.00	0.00, 0.12 (j = 1/2, j = 3/2)
^2E	$a_1^2\, e^3\, t_2^6\, t_1^0\, t_2^0$ (54%)	1.12	1.20	1.09	1.16, 1.17
$^4A_2$	$a_1^2\, e^4\, t_2^4\, t_1^1\, t_2^0$ (53%)	1.67	1.74	1.78	1.85
$^2A_1$	$a_1^1\, e^4\, t_2^6\, t_1^0\, t_2^0$ (51%)	1.84	1.92	1.69	1.77
^4E	$a_1^2\, e^4\, t_2^4\, t_1^1\, t_2^0$ (46%)	2.04	2.09 - 2.11	2.02	2.07 - 2.09
$^4T_1$	$a_1^2\, e^4\, t_2^4\, t_1^1\, t_2^0$ (44%)	2.12	2.19 - 2.22	2.08	2.15 - 2.18
$^4T_1$	$a_1^2\, e^4\, t_2^4\, t_1^1\, t_2^0$ (40%)	2.20	2.26 - 2.29	2.15	2.21 - 2.23
$^2T_1$	$a_1^2\, e^4\, t_2^4\, t_1^1\, t_2^0$ (43%)	2.24	2.34, 2.35	2.24	2.34, 2.34
$^2T_1$	$a_1^2\, e^4\, t_2^4\, t_1^1\, t_2^0$ (43%)	2.32	2.41, 2.42	2.27	2.35, 2.37
^2E	$a_1^2\, e^4\, t_2^4\, t_1^1\, t_2^0$ (39%)	2.43	2.52	2.21	2.31

In $GaMo_4Se_8$, the effect of SOC is of similar magnitude, since it mainly originates from the 4*d* orbitals of Mo. Generally, the low-energy excitation spectrum yields lower values by about 50 to 100 meV for the Se-coordinated lacunar spinel as compared to the S-coordinated one due to longer Mo-Se bond lengths leading to smaller crystal field splittings. Still, the ordering of excited states and the corresponding weight of the leading electronic configuration in the overall wavefunction of each excited state remain close to identical in both compounds, which presumably leads to similar magnetic properties.

Table S2. Low-energy excitation energies (eV) of high-temperature (HT) $[Mo_4Se_{16}]^{19-}$ (CAS(11e,12o)). Four quartets and six doublets were included in the state-averaging procedure. Notation according to T_d point group symmetry.

State	Leading config. (weight)	E_{CASSCF}	$E_{CASSCF+SOC}$	E_{NEVPT2}	$E_{NEVPT2+SOC}$
$^2T_2$	$a_1^2\, e^4\, t_2^5\, t_1^0\, t_2^0$ (62%)	0.00	0.00, 0.13<br>(*j* = 1/2, *j* = 3/2)	0.00	0.00, 0.13<br>(*j* = 1/2, *j* = 3/2)
^2E	$a_1^2\, e^3\, t_2^6\, t_1^0\, t_2^0$ (54%)	1.06	1.14	1.02	1.10
$^4A_2$	$a_1^2\, e^4\, t_2^4\, t_1^1\, t_2^0$ (53%)	1.63	1.71	1.71	1.78
$^2A_1$	$a_1^1\, e^4\, t_2^6\, t_1^0\, t_2^0$ (52%)	1.74	1.82	1.59	1.67
^4E	$a_1^2\, e^4\, t_2^4\, t_1^1\, t_2^0$ (47%)	2.00	2.05 - 2.08	1.95	1.99 - 2.02
$^4T_1$	$a_1^2\, e^4\, t_2^4\, t_1^1\, t_2^0$ (43%)	2.08	2.15 - 2.19	2.00	2.07 - 2.12
$^4T_1$	$a_1^2\, e^4\, t_2^4\, t_1^1\, t_2^0$ (40%)	2.17	2.22 - 2.25	2.07	2.14 - 2.16
$^2T_1$	$a_1^2\, e^4\, t_2^4\, t_1^1\, t_2^0$ (44%)	2.20	2.31, 2.32	2.17	2.27, 2.28
$^2T_1$	$a_1^2\, e^4\, t_2^4\, t_1^1\, t_2^0$ (43%)	2.29	2.38, 2.39	2.20	2.28, 2.31
^2E	$a_1^2\, e^4\, t_2^4\, t_1^1\, t_2^0$ (40%)	2.39	2.49	2.14	2.24

Low-energy excitation energies of low-temperature (LT) phase

For the low-temperature (LT) phases of $GaMo_4S_8$ and $GaMo_4Se_8$ (Tables S3 and S4), triply degenerate T_1/T_2 states are split into A_2/A_1 and E terms as a result of the Jahn-Teller distortion. This effect is more pronounced as compared to the spin-orbit splitting in the HT phase (~300 vs. ~100 meV). Since SOC is not active for the $^2A_1$ ground state, the structure adopts an effective spin-1/2 under the influence of Jahn-Teller distortion. Interestingly, the underlying $a_1^2\, e^4\, e^4\, a_1^1$ leading configuration of this state comes with a weight of only 62%, comparable to the $^2T_2$ ground state at high temperatures. Also, excited states show a decreasing weight of the leading configuration since more configurations mix into the overall wavefunction. By correcting the CASSCF values for dynamical correlation with the NEVPT2 approach a strong decrease in the excited state energies is observed for the $S = 1/2$ and most of $S = 3/2$ states. Still, there is some re-ordering of the excited state terms observed at ~2 eV, e.g. involving $^2A_1$ and ^4E states.

Table S3. Low-energy excitation energies (eV) of low-temperature (LT) $[Mo_4S_{16}]^{19-}$ (CAS(11e,12o)). Nine quartets and fifteen doublet roots were included in the state-averaging procedure. Notation according to C_{3v} point group symmetry.

State	Leading config. (weight)	E_{CASSCF}	$E_{CASSCF+SOC}$	E_{NEVPT2}	$E_{NEVPT2+SOC}$
$^2A_1$	$a_1^2\ e^4\ e^4\ a_1^1\ a_2^0\ e^0\ e^0\ a_1^0$ (62%)	0.00	0.00	0.00	0.00
^2E	$a_1^2\ e^4\ e^3\ a_1^2\ a_2^0\ e^0\ e^0\ a_1^0$ (61%)	0.41	0.39, 0.45	0.33	0.32, 0.38
^2E	$a_1^2\ e^3\ e^4\ a_1^2\ a_2^0\ e^0\ e^0\ a_1^0$ (52%)	1.36	1.37, 1.37	1.29	1.30, 1.31
$^4A_2$	$a_1^2\ e^4\ e^3\ a_1^1\ a_2^0\ e^1\ e^0\ a_1^0$ (37%)	1.94	1.94, 1.94	2.01	2.00
^4E	$a_1^2\ e^4\ e^3\ a_1^1\ a_2^1\ e^0\ e^0\ a_1^0$ (36%)	2.10	2.10 - 2.11	2.08	2.08 - 2.09
$^2A_1$	$a_1^1\ e^4\ e^4\ a_1^2\ a_2^0\ e^0\ e^0\ a_1^0$ (47%)	2.16	2.17	2.00	2.01
^2E	$a_1^2\ e^4\ e^4\ a_1^0\ a_2^0\ e^1\ e^0\ a_1^0$ (23%)	2.27	2.26, 2.27	2.16	2.16, 2.17
^4E	$a_1^2\ e^4\ e^3\ a_1^1\ a_2^0\ e^1\ e^0\ a_1^0$ (34%)	2.37	2.36 - 2.38	2.25	2.25 - 2.27
$^4A_2$	$a_1^2\ e^4\ e^3\ a_1^1\ a_2^0\ e^1\ e^0\ a_1^0$ (49%)	2.37	2.39, 2.39	2.25	2.28, 2.28
$^4A_2$	$a_1^2\ e^4\ e^2\ a_1^2\ a_2^1\ e^0\ e^0\ a_1^0$ (30%)	2.43	2.44	2.42	2.42, 2.44
$^2A_2$	$a_1^2\ e^4\ e^3\ a_1^1\ a_2^0\ e^1\ e^0\ a_1^0$ (33%)	2.49	2.50	2.44	2.45
^2E	$a_1^2\ e^4\ e^3\ a_1^1\ a_2^0\ e^1\ e^0\ a_1^0$ (30%)	2.55	2.56, 2.57	2.45	2.46, 2.48
$^2A_2$	$a_1^2\ e^4\ e^4\ a_1^0\ a_2^1\ e^0\ e^0\ a_1^0$ (36%)	2.56	2.58	2.38	2.40
$^2A_2$	$a_1^2\ e^4\ e^3\ a_1^1\ a_2^0\ e^1\ e^0\ a_1^0$ (32%)	2.62	2.63	2.41	2.41
^4E	$a_1^2\ e^4\ e^2\ a_1^2\ a_2^0\ e^1\ e^0\ a_1^0$ (23%)	2.67	2.68 - 2.69	2.60	2.61 - 2.62
^2E	$a_1^2\ e^4\ e^3\ a_1^1\ a_2^1\ e^0\ e^0\ a_1^0$ (26%)	2.82	2.82, 2.84	2.55	2.56, 2.57

Table S4. Low-energy excitation energies (eV) of low-temperature (LT) $[Mo_4Se_{16}]^{19-}$ (CAS(11e,12o)). Nine quartets and fifteen doublet roots were included in the state-averaging procedure. Notation according to C_{3v} point group symmetry.

State	Leading config. (weight)	E_{CASSCF}	$E_{CASSCF+SOC}$	E_{NEVPT2}	$E_{NEVPT2+SOC}$
$^2A_1$	$a_1^2\ e^4\ e^4\ a_1^1\ a_2^0\ e^0\ e^0\ a_1^0$ (61%)	0.00	0.00	0.00	0.00
^2E	$a_1^2\ e^4\ e^3\ a_1^2\ a_2^0\ e^0\ e^0\ a_1^0$ (60%)	0.32	0.30, 0.37	0.24	0.23, 0.29
^2E	$a_1^2\ e^3\ e^4\ a_1^2\ a_2^0\ e^0\ e^0\ a_1^0$ (52%)	1.25	1.26, 1.27	1.17	1.18
$^4A_2$	$a_1^2\ e^4\ e^3\ a_1^1\ a_2^0\ e^1\ e^0\ a_1^0$ (28%)	1.78	1.78, 1.79	1.80	1.80, 1.81
$^2A_1$	$a_1^1\ e^4\ e^4\ a_1^2\ a_2^0\ e^0\ e^0\ a_1^0$ (51%)	1.91	1.91	1.72	1.73
^4E	$a_1^2\ e^4\ e^3\ a_1^1\ a_2^1\ e^0\ e^0\ a_1^0$ (41%)	1.92	1.92 - 1.93	1.86	1.86 - 1.88
^2E	$a_1^2\ e^4\ e^3\ a_1^1\ a_2^1\ e^1\ e^0\ a_1^0$ (26%)	2.14	2.12, 2.14	2.03	2.01, 2.03
^4E	$a_1^2\ e^4\ e^3\ a_1^1\ a_2^0\ e^1\ e^0\ a_1^0$ (34%)	2.22	2.22 - 2.24	2.06	2.07 - 2.10
$^4A_2$	$a_1^2\ e^4\ e^3\ a_1^1\ a_2^0\ e^1\ e^0\ a_1^0$ (48%)	2.24	2.25, 2.26	2.10	2.12, 2.12
$^4A_2$	$a_1^2\ e^4\ e^2\ a_1^2\ a_2^1\ e^0\ e^0\ a_1^0$ (23%)	2.26	2.27, 2.29	2.17	2.19, 2.20
^2E	$a_1^2\ e^4\ e^3\ a_1^1\ a_2^0\ e^1\ e^0\ a_1^0$ (23%)	2.39	2.40, 2.41	2.22	2.24, 2.25
$^2A_2$	$a_1^2\ e^4\ e^4\ a_1^0\ a_2^0\ e^1\ e^0\ a_1^0$ (26%)	2.40	2.42	2.25	2.27
$^2A_2$	$a_1^2\ e^4\ e^3\ a_1^1\ a_2^0\ e^1\ e^0\ a_1^0$ (33%)	2.40	2.43	2.29	2.30
$^2A_2$	$a_1^2\ e^4\ e^3\ a_1^1\ a_2^0\ e^1\ e^0\ a_1^0$ (22%)	2.44	2.46	2.17	2.17
^4E	$a_1^2\ e^4\ e^2\ a_1^2\ a_2^0\ e^1\ e^0\ a_1^0$ (27%)	2.58	2.59 - 2.60	2.40	2.42 - 2.43
^2E	$a_1^2\ e^4\ e^3\ a_1^1\ a_2^1\ e^0\ e^0\ a_1^0$ (27%)	2.68	2.68, 2.71	2.33	2.35, 2.37

SM-5. Susceptibility Measurements on $GaMo_4S_8$

Temperature-dependent magnetic susceptibility curves across the structural transition were measured for single-crystalline $GaMo_4S_8$ using a SQUID magnetometer (Quantum Design MPMS3) along the cubic [100] direction with a magnetic field of 3 T (see Figure S2). The phase transition is clearly visible as a kink in the inverse susceptibility at T_s = 45 K. A Curie-Weiss fit of the inverse susceptibility in the cubic high-temperature phase yields an effective paramagnetic moment of μ_{eff} = 2.05 μB and a Curie-Weiss temperature Θ = 0.22 K. This effective paramagnetic moment agrees well with the one found by Powell *et al.*[3] Besides, using the relation $g=\mu_{eff}/\sqrt{S(S+1)}$ and a spin of S = 1/2, a measured g factor of g_{exp} = 2.37 is obtained, which exceeds the calculated one of g_{calc} = 2.18 for $[Mo_4S_{16}]^{19-}$ (see manuscript). This deviation can be attributed to vibronic effects, that are not included in our calculations due to a rigid cluster model. At temperatures above 100 K, this is also reflected by the difference in the measured and calculated susceptibility data shown in Figure S2. Similar observations were also made for the related lacunar spinels $GaNb_4Se_8$ and $GaTa_4Se_8$.[2]

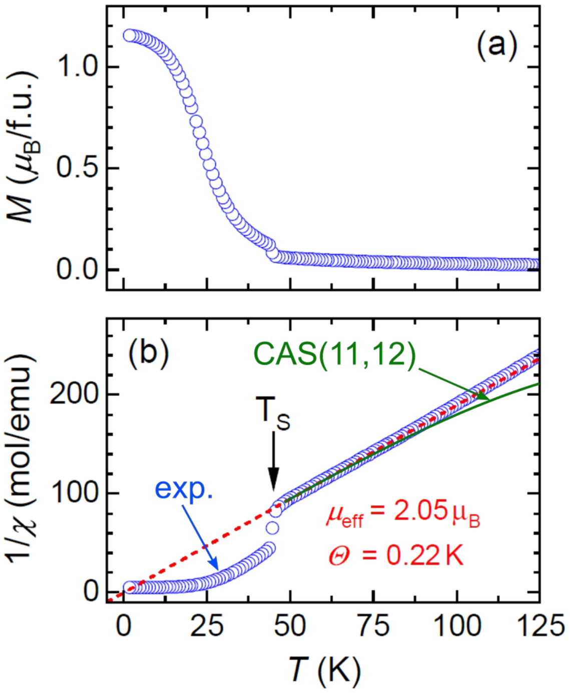

Figure S2. Susceptibility measurements on $GaMo_4S_8$. (a) Temperature-dependent magnetization of $GaMo_4S_8$ measured at 3 T along the cubic [100] direction. (b) Corresponding inverse susceptibility 1/χ. The dashed, red line represents the Curie-Weiss fitted line for the cubic high-temperature data. The solid, green line refers to the calculated magnetic susceptibility using the $[Mo_4S_{16}]^{19-}$/CAS(11e,12o) embedded cluster approach.

SM-6. Different Low-Temperature (LT) $GaMo_4S_8$ Structures

In the manuscript, the crystal structure data of $GaMo_4S_8$ was taken from Powell *et al.*[3] Since deviations in the bond lengths/angles with respect to the measured crystal cannot be excluded, their effect on magnetic properties was studied. For this, we adopt the LT-$GaMo_4S_8$ crystal structure proposed by Francois *et al.*,[25] which shows slightly longer Mo-Mo bond lengths of about 0.04 Å. The associated low-energy excitation energies are given in Table S5. Compared to the structure of Powell *et al.*[3] (see Table S3), the energies in Table S5 are considerably lower. This can be explained by the longer bonds of the latter, resulting in a decreased crystal field splitting in the Mo_4 tetrahedron.

Associated with the decreased SOC eigenvalues in Table S5, the g factor becomes more anisotropic: for a CAS(11e,12o) we obtain $g_{\parallel} = 1.58$ and $g_{\perp} = 2.67$ using NEVPT2 ($g_{\parallel} = 1.71$ and $g_{\perp} = 2.59$ using CASSCF). As a consequence, the simulated magnetization curves (see Figure S4) also differ from the ones presented in Figure 2 of the manuscript. Most evident here is the lower $[1\bar{1}0]$ saturation magnetization value by about 0.07 μ_B, which still differs by 0.03 μ_B from the experimentally measured curve. From a theoretical perspective, it is therefore difficult to reach overall quantitative agreement, since slight deviations in the structure can be expected based on the experimental set-up.

Table S5. Low-energy excitation energies (eV) of low-temperature (LT) $[Mo_4S_{16}]^{19-}$ using the geometric structure proposed by Francois *et al.*[25] (CAS(11e,12o)).

State	Leading config. (weight)	E_{CASSCF}	$E_{CASSCF+SOC}$	E_{NEVPT2}	$E_{NEVPT2+SOC}$
$^2A_1$	$a_1^2\ e^4\ e^4\ a_1^1\ a_2^0\ e^0\ e^0\ a_1^0$ (61%)	0.00	0.00	0.00	0.00
^2E	$a_1^2\ e^4\ e^3\ a_1^2\ a_2^0\ e^0\ e^0\ a_1^0$ (60%)	0.28	0.27, 0.33	0.24	0.23, 0.29
^2E	$a_1^2\ e^3\ e^4\ a_1^2\ a_2^0\ e^0\ e^0\ a_1^0$ (51%)	1.23	1.24, 1.24	1.19	1.20, 1.20
$^4A_2$	$a_1^2\ e^4\ e^3\ a_1^1\ a_2^0\ e^1\ e^0\ a_1^0$ (31%)	1.74	1.75, 1.75	1.83	1.83, 1.83
^4E	$a_1^2\ e^4\ e^3\ a_1^1\ a_2^1\ e^0\ e^0\ a_1^0$ (36%)	1.93	1.93 - 1.95	1.92	1.93 - 1.94
$^2A_1$	$a_1^1\ e^4\ e^4\ a_1^2\ a_2^0\ e^0\ e^0\ a_1^0$ (48%)	1.94	1.95	1.79	1.80
^2E	$a_1^2\ e^4\ e^3\ a_1^1\ a_2^1\ e^0\ e^0\ a_1^0$ (23%)	2.14	2.12, 2.13	2.08	2.06, 2.07
^4E	$a_1^2\ e^4\ e^3\ a_1^1\ a_2^0\ e^1\ e^0\ a_1^0$ (32%)	2.17	2.17 - 2.20	2.09	2.10 - 2.13
$^4A_2$	$a_1^2\ e^4\ e^3\ a_1^1\ a_2^0\ e^1\ e^0\ a_1^0$ (45%)	2.20	2.20, 2.21	2.12	2.15, 2.16
$^4A_2$	$a_1^2\ e^4\ e^2\ a_1^2\ a_2^1\ e^0\ e^0\ a_1^0$ (25%)	2.20	2.23, 2.25	2.20	2.21, 2.22
$^2A_2$	$a_1^2\ e^4\ e^3\ a_1^1\ a_2^0\ e^1\ e^0\ a_1^0$ (33%)	2.32	2.33	2.30	2.31
^2E	$a_1^2\ e^4\ e^3\ a_1^1\ a_2^0\ e^1\ e^0\ a_1^0$ (23%)	2.35	2.36, 2.37	2.26	2.27, 2.29
$^2A_2$	$a_1^2\ e^4\ e^3\ a_1^1\ a_2^0\ e^1\ e^0\ a_1^0$ (29%)	2.38	2.40	2.31	2.34
^4E	$a_1^2\ e^4\ e^2\ a_1^2\ a_2^0\ e^1\ e^0\ a_1^0$ (26%)	2.45	2.46 - 2.47	2.39	2.40 - 2.41
$^2A_2$	$a_1^2\ e^4\ e^4\ a_1^0\ a_2^1\ e^0\ e^0\ a_1^0$ (37%)	2.46	2.48	2.21	2.24
^2E	$a_1^2\ e^4\ e^2\ a_1^2\ a_2^0\ e^1\ e^0\ a_1^0$ (18%)	2.64	2.65, 2.67	2.49	2.51, 2.53

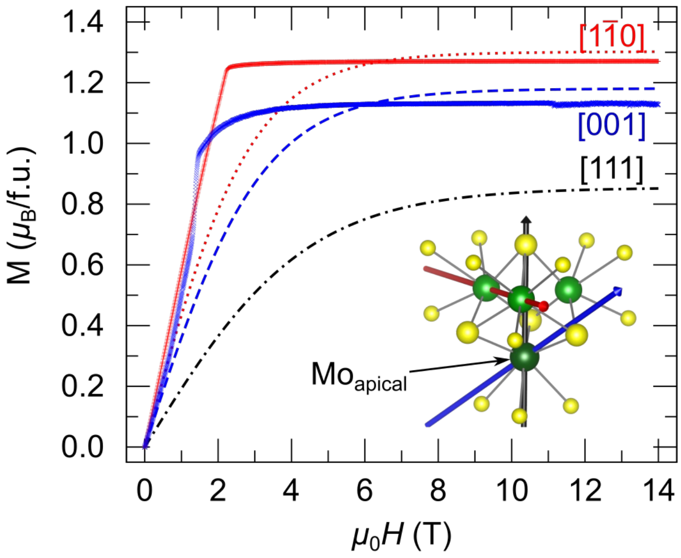

Figure S3. Magnetization curves for LT $GaMo_4S_8$. Both measured (full lines) and simulated (dashed lines) at 2.5 K are shown (CASSCF(11e,12o)). The simulated curves are obtained for LT $[Mo_4S_{16}]^{19-}$ using crystal parameters of Francois *et al.*[25]

SM-7. ORCA Input

The coordinates for the $[Mo_4S_{16}]^{19-}$ and $[Mo_4Se_{16}]^{19-}$ embedded cluster models were extracted from experimentally determined structures for the high-temperature $F\bar{4}3m$ and low-temperature $R3m$ phase of both compounds.[3,4,25] All coordinates are given in separate spreadsheets. Additionally, an input example for a CASSCF+NEVPT2+SOC calculation of $[Mo_4S_{16}]^{19-}$ in ORCA5.0 is given in the following Figure S4. In this particular example, the discussed high-temperature excited states (fourteen doublet and nine quartet roots) are requested in the CASSCF block as well as a subsequent treatment of SOC.

```
!DKH CASSCF RIJCOSX RI-NEVPT2 NOFROZENCORE

%rel
  picturechange 2
  fpFWtrafo false
end

%casscf
  nel 11
  norb 12
  mult 4,2
  nroots 9,14
  bweight 9,14
  ci
   maxiter 500
  end
  ptsettings
   d4step efficient
   d4tpre 1e-14
  end
  rel
    dosoc true
    gtensor true
  end
  aniso
    doaniso true
    mltp 2,4
  end
end

%basis
  NewGTO Mo “SARC-DKH-TZVPP” end
  NewGTO S “DKH-DEF2-TZVPP” end
  NewAuxJGTO Mo “AUTOAUX” end
  NewAuxCGTO Mo “AUTOAUX” end
  NewAuxJGTO S “DEF2/J” end
  NewAuxCGTO S “DEF2-TZVPP/C” end
  DecontractAuxJ true
  DecontractAuxC true
end

%pointcharges “pointcharges.pc”

*xyz -19 2
  ...
*
```

Figure S4. Orca input example. This example is used for an embedded cluster calculation of $[Mo_4S_{16}]^{19-}$ using CASSCF+NEVPT2+SOC. An SA-CAS(11e,12o) solution over the lowest nine quartet and 14 doublet roots is requested. Coordinates are given in the separate spreadsheet.

Supplementary References

1 Pocha, R., Johrendt, D. & Pöttgen, R. Electronic and Structural Instabilities in GaV_4S_8 and $GaMo_4S_8$. *Chem. Mater.* **12**, 2882 (2000).

2 Petersen, T., Prodan, L., Tsurkan, V., Krug von Nidda, H.-A., Kézsmárki, I., Rößler, U. K. & Hozoi, L. How Correlations and Spin-Orbit Coupling Work within Extended Orbitals of Transition-Metal Tetrahedra of 4d/5d Lacunar Spinels. *J. Phys. Chem. Lett.* **13**, 1681 (2022).

3 Powell, A. V., McDowall, A., Szkoda, I. Knight, K. S., Kennedy, B. J. & Vogt, T. Cation Substitution in Defect Thiospinels: Structural and Magnetic Properties of $GaV_{4-x}Mo_xS_8$ ($0 \le x \le 4$). *Chem. Mater.* **19**, 5035 (2007).

4 Routledge, K., Vir, P., Cook, N., Murgatroyed, P. A. E., Ahmed, S. J., Savvin, S. N., Claridge, J. B. & Alaria, J. Mode crystallography analysis through the structural phase transition and magnetic critical behavior of the lacunar spinel $GaMo_4Se_8$. *Chem. Mater.* **33**, 5718 (2021).

5 Klintenberg, M., Derenzo, S. & Weber, M. Accurate crystal fields for embedded cluster calculations. *Comp. Phys. Commun.* **131**, 120 (2000).

6 Derenzo, S. E., Klintenberg, M. K. & Weber, M. J. Determining point charge arrays that produce accurate ionic crystal fields for atomic cluster calculations. *J. Chem. Phys.* **112**, 2074 (2000).

7 Breneman, C. M. & Wiberg, K. B. Determining atom-centered monopoles from molecular electrostatic potentials. The need for high sampling density in formamide conformational analysis. *J. Comput. Chem.* **11**, 361 (1990).

8 Dittmer, A., Stoychev, G. L., Maganas, D., Auer, A. A. & Neese, F. Computation of NMR Shielding Constants for Solids Using an Embedded Cluster Approach with DFT, Double-Hybrid DFT, and MP2. *J. Chem. Theor. Comput.* **16**, 6950 (2020).

9 Helgaker, T., Jorgensen, P. & Olsen, J. *Molecular Electronic-Structure Theory* (Wiley VCH, Chichester, 2000).

10 Angeli, C., Cimiraglia, R., Evangelisti, S., Leininger, T. & Malrieu, J.-P. Introduction of N-electron valence states for multireference perturbation theory. *J. Chem. Phys.* **114**, 10252 (2001).

11 Dunning, T. H. Gaussian basis sets for use in correlated molecular calculations. I. The atoms boron through neon and hydrogen. *J. Chem. Phys.* **90**, 1007 (1989).

12 Rolfes, J. D., Neese, F. & Pantazis, D. A. All-electron scalar relativistic basis sets for the elements Rb–Xe. *J. Comput. Chem.* **41**, 1842 (2020).

13 Weigend, F. & Ahlrichs, R. Balanced basis sets of split valence, triple zeta valence and quadruple zeta valence quality for H to Rn: Design and assessment of accuracy. *Phys. Chem. Chem. Phys.* **7**, 3297 (2005).

14 Stoychev, G. L., Auer, A. A. & Neese, F. Automatic generation of auxiliary basis sets. *J. Chem. Theor. Comput.* **13**, 554 (2017).

15 Weigend, F. Accurate coulomb-fitting basis sets for H to Rn. *Phys. Chem. Chem. Phys.* **8**, 1057 (2006).

16 Hellweg, A. Hättig, C., Höfener, S. & Klopper, W. Optimized accurate auxiliary basis sets for RI-MP2 and RI-CC2 calculations for the atoms Rb to Rn. *Theor. Chem. Acc.*

117, 587 (2007).

17 Andrae, D., Häusermann, U., Dolg, M., Stoll, H. & Preus, H. Energy-adjusted ab initiopseudopotentials for the second and third row transition elements. *Theoret. Chim. Acta* **77**, 123 (1990).

18 Bergner, A., Dolg, M., Küchle, W., Stoll, H. & Preus, H. Ab initio energy-adjusted pseudopotentials for elements of groups 13–17. *Mol. Phys.* **80**, 1431 (1993).

19 Leininger, T., Berning, A., Nicklass, A., Stoll, H. Werner, H.-J. & Flad, H.-J. Spin-orbit interaction in heavy group 13 atoms and TlAr. *Chem. Phys.* **217**, 19 (1997).

20 Neese, F. Software update: The ORCA program system—Version 5.0. *WIREs Comput. Mol. Sci.* **12**, e1606 (2022).

21 Becke, A. D. Density-functional exchange-energy approximation with correct asymptotic behavior. *Phys. Rev. A* **38**, 3098 (1988).

22 Lee, C., Yang, W. & Parr, R. G. Development of the Colle-Salvetti correlation-energy formula into a functional of the electron density. *Phys. Rev. B* **37**, 785 (1988).

23 Vosko, S. H., Wilk, L. & Nusair, M. Accurate spin-dependent electron liquid correlation energies for local spin density calculations: a critical analysis. *Can. J. Phys.* **58**, 1200 (1980).

24 Stephens, P. J., Devlin, F. J., Chabalowski, C. F. & Frisch, M. J., Ab Initio Calculation of Vibrational Absorption and Circular Dichroism Spectra Using Density Functional Force Fields. *J. Phys. Chem.* **98**, 11623 (1994).

25 Francois, M., Lengauer, W., Yvon, K., Sergent, M., Potel, M., Gougeon, P. & Ben Yaich-Aerrache, H. Structural phase transition in $GaMo_4S_8$ by x-ray powder diffraction. *Z. Kristallogr.* **196**, 111 (1991).
